# Assessment of Rib Fracture in Acute Trauma Using Automatic Rib Segmentation and a Curved, Unfolded View of the Ribs: Is There a Saving of Time?

**DOI:** 10.3390/jcm11092502

**Published:** 2022-04-29

**Authors:** Benedikt Pregler, Lukas Philipp Beyer, Natascha Platz Batista da Silva, Sebastian Steer, Florian Zeman, Daniel Popp, Christian Stroszczynski, René Müller-Wille

**Affiliations:** 1 Department of Radiology, Ernst von Bergmann Klinikum Potsdam, 14467 Potsdam, Germany; lukas.beyer@klinikumevb.de; 2 Department of Radiology, University Medical Center Regensburg, 93053 Regensburg, Germany; natascha.platz@ukr.de (N.P.B.d.S.); sebastian.steer@ukr.de (S.S.); christian.stroszczynski@ukr.de (C.S.); 3 Center for Clinical Studies, University Medical Center Regensburg, 93053 Regensburg, Germany; florian.zeman@ukr.de; 4 Department of Trauma Surgery, University Medical Center Regensburg, 93053 Regensburg, Germany; daniel.popp@ukr.de; 5 Department of Radiology, Klinikum Wels-Grieskirchen, 4600 Wels, Austria; rene.mueller-wille@ukr.de

**Keywords:** radiology, polytrauma, rib fracture, diagnosis, segmentation, sensitivity and specificity, computed tomography

## Abstract

Introduction: The fast and accurate diagnosis of rib fractures in polytrauma patients is important to reduce the mortality rate and relieve long-term pain and complications. Aim: To evaluate the diagnostic accuracy and potential time savings using automatic rib segmentation and a curved, unfolded view for the detection of rib fractures in trauma patients. Methods: The multidetector computed tomography raw data of 101 consecutive polytrauma patients (72 men; mean age 45 years, age range 17 to 84 years) admitted to a university hospital were retrospectively post-processed to generate a curved, unfolded view of the rib cage. No manual corrections were performed. Patients with reconstruction errors and movement artifacts were excluded from further analysis. All fractures were identified and classified by the study coordinator using the original data set. Two readers (reader 1 and reader 2) evaluated the original axial sections and the unfolded view, separately. The fracture locations, fracture type, and reading times were recorded. Sensitivity and specificity were calculated on a per-rib basis using a ratio estimator. Cohen’s Kappa was calculated as an index of inter-rater agreement. Results: 26 of 101 patients (25.7%) were excluded from further analysis owing to breathing artifacts (6.9%) or incorrect centerline computation in the unfolded view (18.8%). In total, 107 (5.9%) of 1800 ribs were fractured in 25 (33%) of 75 patients. The unfolded view had a sensitivity/specificity of 81%/100% (reader 1) and 71%/100% (reader 2) compared to 94%/100% (reader 1; *p* = 0.002/*p* = 0.754) and 63%/99% (reader 2; *p* < 0.001/*p* = 0.002). The sensitivity (reader 1; reader 2) was poor for buckled fractures (31%; 38%), moderate for undislocated fractures (78%; 62%), and good for dislocated fractures (94%; 90%). The assessment of the unfolded view was performed significantly faster than that of the original layers (19.5 ± 9.4 s vs. 68.6 ± 32.4 s by reader 1 (*p* < 0.001); 24.1 ± 9.5 s vs. 40.2 ± 12.7 s by reader 2 (*p* < 0.001)). Both readers demonstrated a very high interobserver agreement for the unfolded view (κ = 0.839) but only a moderate agreement for the original view (κ = 0.529). Conclusion: Apart from a relatively high number of incorrect centerline reconstructions, the unfolded view of the rib cage allows a faster diagnosis of dislocated rib fractures.

## 1. Introduction

A recent meta-analysis has demonstrated that polytrauma patients with a recent fracture of three or more ribs have an increased mortality risk [1]. Serial rib fractures always reflect the severity of the chest trauma, and they are associated with life-threatening complications such as pneumothorax, hemothorax, and pulmonary contusions [2,3]. Recent studies have also shown that the contribution of rib fractures to prolonged chest pain and disability is greater than traditionally thought [4,5]. Therefore, the fast and accurate diagnosis of rib fractures in polytrauma patients is important to reduce the mortality rate and relieve long-term pain and complications.

Modern whole-body computer tomography plays a key role in the emergency diagnosis of trauma patients and significantly increases the likelihood of survival for these patients [6,7,8]. An accurate assessment of the ribs using multi-detector computed tomography (MDCT), particularly in the commonly used axial reconstructions, is frequently difficult owing to the downward sloping orientation of the ribs. The successive, slice-by-slice examination of each individual rib on axial slices is very time-consuming and error-prone. Time pressure and a noisy environment, which are common during the examination of trauma patients, lead to increased error rates [9,10].

Automated segmentation methods including auxiliary algorithms such as the “unfolding” of complex anatomy structures have been invented with the aim of increasing diagnostic speed and accuracy. For example, for CT colonography [11] or imaging of the skull [12,13], it has been shown that assessment can be simplified and sensitivity and specificity increased by using advanced segmentation-based visualization techniques. To transfer this visualization technique to the assessment of rib fractures, new software for automatic rib segmentation and advanced visualization has been developed [14,15]. After automatic segmentation, a multiplanar reconstruction along the midlines of the ribs is performed in the so-called “unfolded” view; all ribs can thus be displayed seamlessly in one image without time-consuming scrolling.

Although previous studies have demonstrated high diagnostic accuracy for this technique [14,15], manual post-processing was performed in all studies when fully automated centerline detection failed. This manual post-processing can sometimes be very time-consuming, limiting its utility in a polytrauma setting where time is of the essence. Studies investigating in which cases automatic reconstruction is successful and limiting further evaluation to these cases have not existed. Likewise, there have been no studies investigating the influence of the experience of the reader on the additional diagnostic benefit.

Therefore, the aim of the present study was to evaluate the diagnostic accuracy and potential time savings of using the unfolded view for the detection of rib fractures in a realistic polytrauma setting, taking into account the reader’s experience.

## 2. Materials and Methods

### 2.1. Study Design and Patient Selection

The retrospective single-center study was approved by the Ethics Committee of the University Regensburg (approval number 18-957-104). It was performed in accordance with the relevant guidelines and regulations and informed consent was waived.

The MDCT raw data of 101 consecutive polytrauma patients who were admitted to our university hospital were retrospectively post-processed using dedicated rib-unfold software (Syngo.via CT-Bone-Reading, Siemens, Erlangen, Germany).

### 2.2. CT Acquisition and Post-Processing

All MDCT scans were performed with a 2 × 128-row CT scanner (SOMATOM Definition Flash, Siemens Healthcare, Forchheim, Germany) using our routine protocol for polytrauma patients: Tube voltage 120 kV, tube current 230 quality reference mAs (CARE Dose, Siemens, Erlangen, Germany). Axial reconstructions of the thorax were created with a section thickness and interval of 5 mm using the B60f kernel for bone and lung tissues and the B31f kernel for soft tissues and vessels. The investigation was performed with a delay of 55 s after the injection of 120 mL Accupaque 350 (GE Healthcare Buchler, Braunschweig, Germany) as a contrast medium at a flow rate of 3 mL/s.

**Figure 1:**
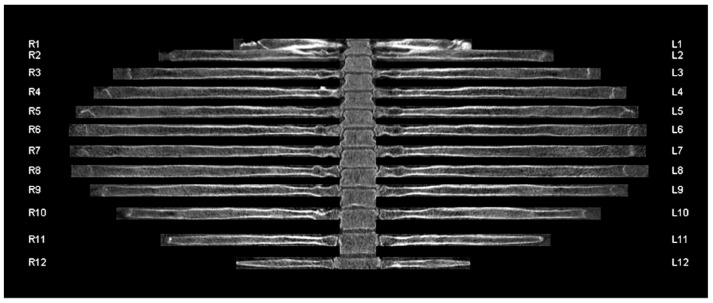
Unfolded rib view of a 41-year-old male patient involved in a high-speed motor vehicle accident with no evidence of acute trauma injury. The curved view and anatomical labels were automatically computed by the reconstruction algorithm. R1-12 refers to the 1st to 12th rib of the right hemithorax. L1-12 refers to the 1st to 12th rib of the left hemithorax.

The raw data of the contrast-enhanced MDCT polytrauma scans were post-processed using the CT-Bone-Reading workflow of Syngo.via. Centerlines through the course of the ribs and anatomical labels were automatically computed by the reconstruction algorithm (Figure 1). A curved (“unfolded”) 2D view of the rib cage was generated based on the centerlines and archived in our PACS system as a series of radial rib ranges (10° intervals, ranging from 0° to 180°). The reconstruction was automatically performed on an external workstation without user input and required approximately one minute per patient dataset. No manual correction of centerlines was performed in case of reconstruction errors.

### 2.3. Image Analysis

**Figure 2:**
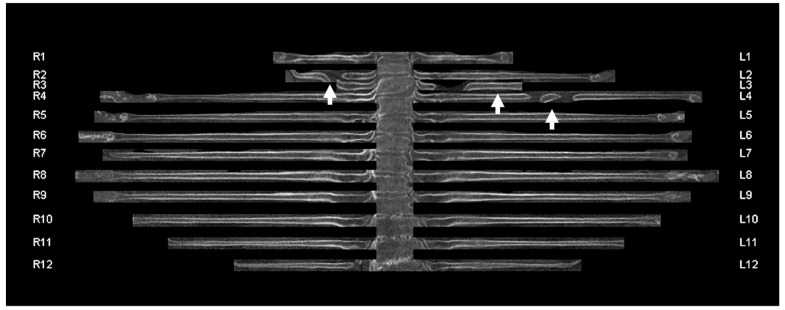
Unfolded rib view of an 83-year-old female patient with reconstruction errors. The centerlines of three ribs were not correctly detected by the algorithm (white arrows). R1-12 refers to the 1st to 12th rib of the right hemithorax. L1-12 refers to the 1st to 12th rib of the left hemithorax.

**Figure 3:**
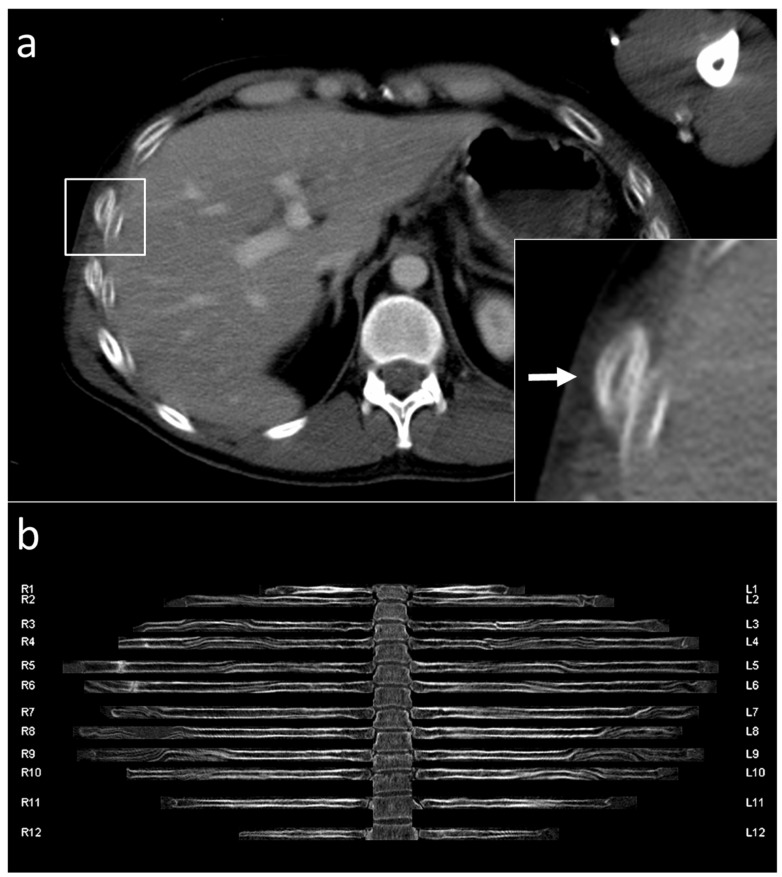
Unfolded rib view of a 33-year-old male patient with breathing artifacts. Typical double-contours (white arrow) in the axial view (**a**) appear like wave shape artifacts in the unfolded view (**b**). R1-12 refers to the 1st to 12th rib of the right hemithorax. L1-12 refers to the 1st to 12th rib of the left hemithorax.

Initially, the series of radial rib ranges were reviewed by the study coordinator (author R.MW., 12 years of experience in radiology) and reviewed for reconstruction errors (Figure 2) and major movement artifacts (Figure 3). Patients with reconstruction errors and artifacts were excluded from further analysis in this study.

Only correctly reconstructed rib thoraces that were free of artifacts were subjected to further analysis by experienced reader 1 (7 years of experience in radiology; author L.P.B.) and inexperienced reader 2 (1 year of experience in radiology; author N.P.). Before the reading, the concept of the “unfolded view” was introduced to both readers. Basic training was done with a training set of 20 patients who were not included in the study set.

**Figure 4:**
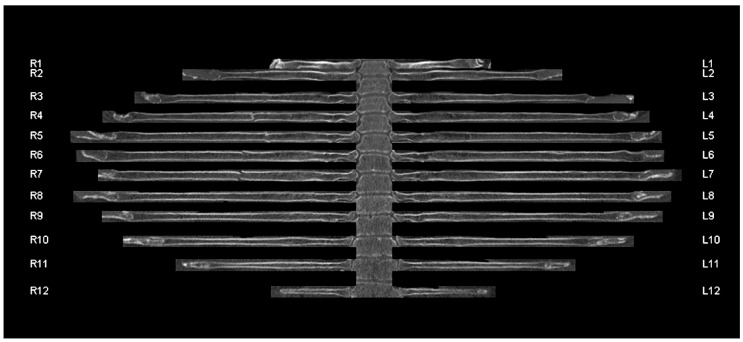
Unfolded rib view of a 64-year-old patient with a serial rib fracture of the 4th to 7th rib on the right side. R1-12 refers to the 1st to 12th rib of the right hemithorax. L1-12 refers to the 1st to 12th rib of the left hemithorax.

For the study, both readers were asked to evaluate the images as they would do under real conditions in a polytrauma setting. They evaluated the original transverse sections and the curved reconstructions separately at an interval of at least 4 weeks. The readings were performed blinded and in randomized order. For each fracture, the side and segment of the fractured rib were documented (Figure 4). Old, consolidated fractures were not graded. A serial rib fracture was defined as a fracture of at least three consecutive ribs. The recording of the fracture locations, patient numbers, and reading times was performed by a postgraduate student (author S.S.), who did not take part as a reader.

**Figure 5:**
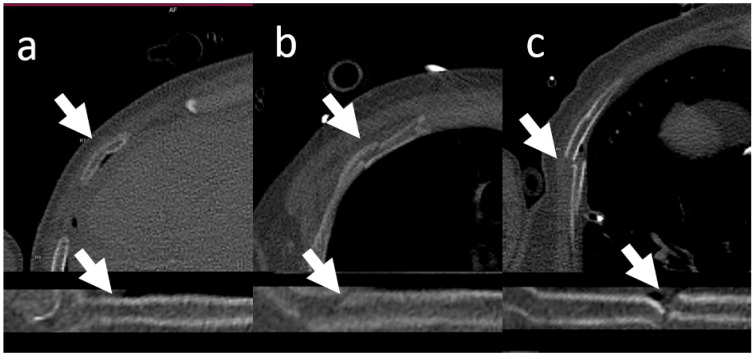
Fracture patterns. Fractures were classified by the study coordinator as incomplete/buckled (**a**), undislocated (**c**), or dislocated (**b**).

To create the reference standard, the original data set was reviewed by the study coordinator (author R.M.-W.). If there was a lack of agreement between the results of the study coordinator and reader 1 and/or 2 (axial sections), a consensus decision was made. All fractures were classified by the study coordinator as incomplete/buckled (discontinuous fracture line), dislocated (offset > ½ bone width), or undislocated (offset ≤ ½ bone width with continuous fracture line; Figure 5).

### 2.4. Statistical Analysis

All analyses were performed using SAS (version 9.4, SAS institute, Cary, NC, USA). The calculation of sensitivities and specificities was performed on a per-rib and a per-patient (no rib fracture versus at least one rib fracture within one patient) basis for the original and unfolded view following standard methodology [16]. The conventional binomial variance estimate assumes that all measurements are independent. Therefore, for rib-based analysis, the patient must be considered as a cluster, as possible patient-based correlations must be considered. Hence, a ratio estimator for the variance of clustered binary data [17] was used to calculate the sensitivity and specificity on a per-rib basis, taking the within-patient correlations into account. Student’s t-test was used to evaluate differences between the reading times. A *p*-value ≤ 0.05 was considered to indicate a significant result. Cohen’s Kappa was calculated as an index of inter-rater agreement [18].

## 3. Results

### 3.1. Segmentation and Labelling

**Figure 6:**
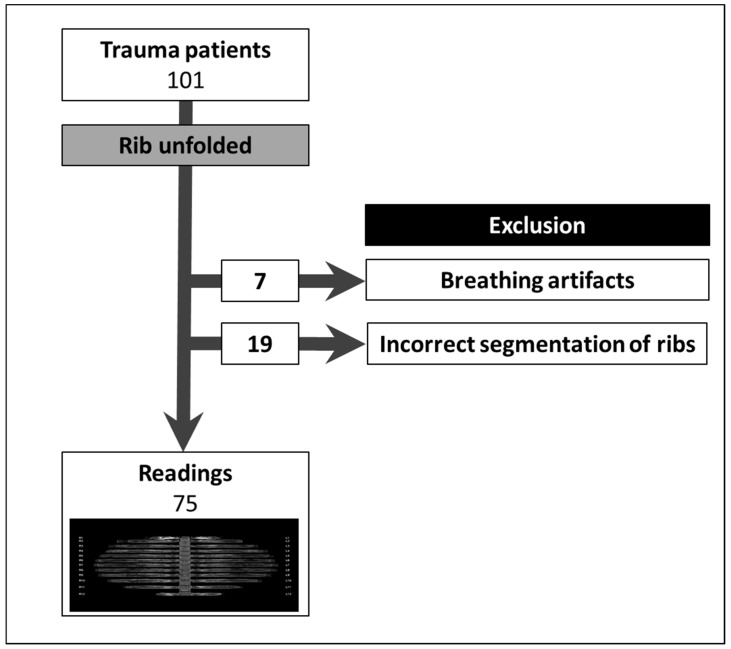
Twenty-six of the 101 patients were excluded. Seven owing to breathing artifacts and 19 owing to the faulty imaging of one or more ribs in the unfolded view.

Overall, 101 consecutive trauma patients were enrolled. Twenty-six patients (25.7%) were excluded from further review either owing to heavy breathing artifacts (7 of 101 (6.9%)) or owing to incorrect centerline computation in the unfolded view (19 of 101 (18.8%); Figure 6). The specific segmentation problems were: 16 centerlines (16 of 2422 ribs (0.7%)) were not detected at all, 52 centerlines (52 of 2422 ribs (2.1%)) were labeled incorrectly (45 up, 7 down). The causes were the incorrect detection of the small thoracic veins filled with contrast agent as ribs and missing 12th ribs (one patient). Stubbed 12th ribs were at times not identified and, in some cases, the transverse processes were labeled as ribs (rarely). In a few cases, the clavicle was labeled as the first rib.

### 3.2. Patient Characteristics

The data from 75 patients (18 women, 57 men, mean age 45 years, age range 17 to 84 years) were available for further analysis. Baseline characteristics are shown in Table 1.

### 3.3. Rib Fractures

Twenty-five of 75 patients (33%) had one or more rib fractures. Fourteen patients (19%) had serial rib fractures. In total, 107 of 1800 ribs (5.9%) were fractured. Of the total, 13 (12.1%) rib fractures were classified as buckled, 63 (58.9%) as undislocated, and 31 (29.0%) as dislocated.

### 3.4. Diagnostic Accuracy

The results of the diagnostic sensitivity and specificity on a per-patient basis and a per-rib basis, ignoring the within-patient correlations, are presented in Table 2. The per-rib results using a ratio estimator for the variance of clustered data to account for repeated measures are shown in Table 3. The unfolded view had a sensitivity/specificity of 81%/100% (reader 1) and 71%/100% (reader 2).

The sensitivity (reader 1; reader 2) was poor for buckled fractures (31%; 38%), moderate for undislocated fractures (78%; 62%) and good for dislocated fractures (94%; 90%) (Table 4). All false-positive findings were caused by minor respiratory motion artifacts misinterpreted as fractures.

Both readers demonstrated a very high interobserver agreement for the unfolded view (κ = 0.807 and 0.839 on a per-patient and per-rib basis, respectively) but only a moderate agreement for the original view (κ = 0.514 and 0.529 on a per-patient and per-rib basis, respectively).

### 3.5. Investigation Time

The assessment of the unfolded view was performed significantly faster than that of the original layers (19.5 ± 9.4 s vs. 68.6 ± 32.4 s, reader 1; 24.1 ± 9.5 s vs. 40.2 ± 12.7 s, reader 2) (Table 5). Interestingly, the inexperienced reader 2 was slightly slower than the experienced reader 1 when examining the unfolded view (difference of 4.6 s). However, when looking at the axial layers, the inexperienced reader 2 was markedly faster (difference of 28.4 s).

## 4. Discussion

The aim of the present study was to investigate whether an unfolded reconstruction of the rib thorax in polytrauma patients can reduce reading times while maintaining or improving diagnostic accuracy for the detection of rib fractures.

This study proposed that using unfolded reconstructions of the rib cage allowed a significant reduction in reading times compared with that of the traditional axial view. The reading times for the tested unfolded view were fast for both the experienced (19.5 s) and inexperienced (24.1 s) readers. The sensitivity and specificity were high for the experienced (81% and 100%) and inexperienced readers (71% and 100%) when using the unfolded view. Regarding the conventional axial view, the inexperienced reader allowed himself substantially less time (40.2 s) than the experienced reader (68.6 s). At the same time, this led to a significantly lower sensitivity (63%) of the inexperienced reader when evaluating the axial images. In the authors’ opinion, the lack of experience led to an underestimation of the care required to thoroughly examine the individual ribs. The lack of experience was probably compensated for by the simpler assessability when reading the multiplanar reformations. Apparently, the inexperienced reader benefits more from the unfolded view.

Rib fracture is one of the most common concomitant injuries in patients with chest trauma [3]. In particular, a serial rib fracture (fracture of at least three or more ribs) is an indicator of the severity of the trauma and is linked to increased mortality [1]. Therefore, the fast and accurate diagnosis of rib fractures constitutes a key diagnostic tool for patients with acute severe trauma.

In a clinical routine, axial layers are first reconstructed in polytrauma because of practical reasons. Traditionally, these are usually considered first and have a comparable specificity and sensitivity to additional fabricated 3D reconstructions that are often used [19]. However, what they both have in common is that they do not allow the entire bony rib skeleton to be viewed briefly. The so-called “unfolded view”, consisting of multiplanar reconstructions along the centerlines of the ribs, is a novel reconstruction method.

In a study by Ringl et al. [14], reading time was also significantly faster when using the unfolded view at 31.6 s compared to 60.7 s using the conventional axial view. However, in contrast to the present study, they performed manual post-processing if the fully automatic detection of the centerlines failed. Because this can sometimes be very time-consuming, the time required for manual correction would have to be added to the reading time to obtain meaningful time information for clinical practice. Therefore, this study was limited to those cases in which the fully automated reconstruction was successful. Their reported sensitivity on a per-rib-basis was similar to this study, with 81.1% for the unfolded view and 76.5% for the axial view.

Bier et al. [16] also reported significant reductions in reading times when using the unfolded view (19.4/26.9/49.9 s vs. 103.7/81.8/154.3 s). In their study, no information is provided on whether and in how many cases manual post-processing was necessary and how long such processing took. This must be considered when interpreting their reading times, which makes it difficult to transfer the results to real-world clinical scenarios. Like the present study, they were able to show that an inexperienced reader benefits more from the unfolded view with a sensitivity of reading the unfolded view at 92.2% vs. 79.7% for the axial view. In the experienced readers, however, there were no significant differences in sensitivity (94.8/94.8% for the unfolded and 93.9/87.9% for the axial view). This could be partially attributed to the fact that the manual tracking of the individual ribs in the axial view is strongly dependent on the experience of the reader.

Khung et al. [20] report a sensitivity of 77% and a specificity of 99% on a per-rib basis when using the unfolded view. In contrast to the other studies and similar to the present study, they did not apply any manual correction. However, there was no reference group for the diagnostic accuracy and the reading times were not reported, which limits the application of their results. Moreover, they did not investigate the differences between experienced and inexperienced readers.

The reconstruction of the unfolded view takes approximately 1 min and runs completely in the background [14]. Because polytrauma scans initially focus on the exclusion of the most severe and immediate life-threatening complications, rib fractures are usually not excluded immediately at the beginning of the examination. Therefore, the exact duration of the reconstruction is likely to be pushed into the background, because the first examination of the images usually exceeds the duration of the reconstruction. However, believing that time-consuming manual correction of the reconstruction in a highly acute environment that requires the quickest possible diagnosis is not appropriate, which is why, unlike previous studies, in this analysis, all patients with reconstruction errors and artifacts were excluded from further evaluation.

While this study was conducted with already-available software in a clinical environment, there are deep learning algorithms in the research field that are capable of automatically detecting fractures. A recent study using a deep-learning algorithm on over 10,000 patients in six hospitals achieved a sensitivity of 85% without and a sensitivity of 89% with human interaction [21]. Another big retrospective single-center study investigated a newly developed deep-learning model for the detection and segmentation of rib fractures, pointing out a high detection sensitivity of 93% without human collaboration and 94 to 96% in the human–computer collaboration cohort [22]. Whether and when these algorithms will find their way into clinical routine is still unclear, but the transfer into diagnostic software will certainly be an exciting field.

A key disadvantage of the investigated method is mainly the high error rate in the automatic detection of the centerlines in the unfolded view (25.7% of patients). While this can be compensated for in clinical routine practice by the conventional viewing of axial images, new segmentation algorithms under development with deep learning neural networks show an accuracy of up to 96% [22]. A manual correction of incomplete or inaccurate rib centerlines, as suggested in other studies, was not performed in our study. This would have been even more time-consuming [14]. To the best of our knowledge, only one other study has been published so far, in which no manual interaction occurred during the reconstruction of the unfolded view [20]. No information on the type and scope of manual correction was reported by Bier et al. [15].

The results of this study are limited by the high number of excluded patients. Moreover, a manual correction of the faulty unfolded bony thoraces was not performed. However, this was a conscious decision within the scope of the study design, because a correction of the segmentation in the case of trauma would take too much time. A manual correction of the centerlines is possible, but this counteracts the advantages of faster reading. In this case, in the authors’ opinion, the axial layers remain the best option for detecting rib fractures.

## 5. Conclusions

In summary, the unfolded view enabled significant time savings in the detection of rib fractures, regardless of the reader’s experience. In terms of diagnostic accuracy, no advantage was noticeable for the experienced reader, whereas the inexperienced reader clearly benefited from the simpler assessability. Therefore, one can conclude that the unfolded view can be a helpful diagnostic tool for the rapid assessment of patients with blunt thorax trauma.

## Figures and Tables

**Table 1:** Baseline characteristics of the 75 patients included in the analysis. Age is given as mean ± standard deviation.

**Characteristics (*n* = 75)**	
Age—years	45 ± 19
Male sex	57 (76%)
**Trauma mechanism**	
Traffic accident	45 (60%)
Fall from great height	24 (32%)
Other	6 (8%)
**Injured regions**	
Head	17 (22.7%)
Chest	35 (46.7%)
Abdomen	23 (30.7%)
Limbs	10 (13.3%)

**Table 2:** Diagnostic accuracy on a per-patient and per-rib basis, not considering repeated measurements. Values given are sensitivity/specificity with the absolute numbers in parentheses.

	Sensitivity		Specificity	
	Axial	Unfolded	Axial	Unfolded
**Per-patient analysis**				
Reader 1	92% (23/25)	84% (21/25)	100% (50/50)	98% (49/50)
Reader 2	56% (14/25)	84% (21/25)	94% (47/50)	96% (48/50)
**Per-rib analysis**				
Reader 1	93% (99/107)	76% (82/107)	100% (1689/1693)	100% (1687/1693)
Reader 2	42% (45/107)	67% (72/107)	99% (1677/1693)	100% (1690/1693)

**Table 3:** Diagnostic accuracy on a per-rib basis considering repeated measures using a ratio estimator for the variance of clustered binary data. Values are given with the 95% confidence interval in square brackets. The *p*-values are calculated ignoring the correlation structure, analyzing the raw data.

	Axial View	Unfolded View	*p*-Value
**Sensitivity**			
Reader 1	94% [90%, 99%]	81% [62%, 100%]	0.002
Reader 2	63% [46%, 81%]	71% [54%, 88%]	<0.001
**Specificity**			
Reader 1	100% [100%, 100%]	100% [100%, 100%]	0.754
Reader 2	99% [99%, 99%]	100% [100%, 100%]	0.002

**Table 4:** Number of true-positive (TP) findings in the unfolded view for different fracture types.

Fracture Type	Buckled	Undislocated	Dislocated
Fractured ribs—no.	13	63	31
Reader 1	4/13 (31%)	49/63 (78%)	29/31 (94%)
Reader 2	5/13 (38%)	39/63 (62%)	28/31 (90%)

**Table 5:** Reading times for the axial and unfolded view and the respective *p*-values. Reading times are given in seconds as mean ± SD (min.—max.).

	Axial View	Unfolded View	*p*-Value
Reader 1	68.6 ± 32.4 (25–157)	19.5 ± 9.4 (8–58)	<0.001
Reader 2	40.2 ± 12.7 (23–101)	24.1 ± 9.5 (10–57)	<0.001

## Data Availability

Not applicable.
